# The Impact of Organic Anion-Transporting Polypeptide (OATP) Variants on the Side Effects of Direct-Acting Antivirals in Hepatitis C Patients

**DOI:** 10.7759/cureus.82213

**Published:** 2025-04-13

**Authors:** Zuhal Altintas, Engin Altintas

**Affiliations:** 1 Medical Genetics, Faculty of Medicine, Mersin University, Mersin, TUR; 2 Gastroenterology and Hepatology, Faculty of Medicine, Mersin University, Mersin, TUR

**Keywords:** direct-acting antiviral agents, gene, hepatitis c virus, oatps, variants

## Abstract

Background

Organic anion-transporting polypeptides (OATPs) are responsible for the cellular uptake of a broad range of endogenous compounds and xenobiotics in multiple tissues. The aim of our study was to determine whether variations in OATP1B1 and OATP1B3 affect the side effects experienced by hepatitis C patients treated with direct-acting antivirals (DAAs).

Methods

This study included 199 hepatitis C patients treated with DAAs. Ledipasvir/sofosbuvir or ombitasvir/paritaprevir/ritonavir ± dasabuvir and 162 control individuals without hepatitis C. Treatment-related side effects were recorded. The OATP1B1 gene variations c.388A>G and c.521T>C and the OATP1B3 gene variations c.334T>G and c.699G>A were analyzed via the polymerase chain reaction-restriction fragment length polymorphism method. Allele/genotype combinations of OATP1B1 and OATP1B3 haplotypes were evaluated.

Results

Side effects were observed in 53 (26.6%) of 199 hepatitis C patients. There were skin mucosal lesions in 19 patients (36%), fatigue in 18 patients (34%), pruritus in 11 patients (20.5%), and other in five patients (9.5%). There was a significant relationship between the c.334T>G variant and side effects (p = 0.030). The frequency distribution of the c.334T>G variant was in Hardy-Weinberg equilibrium. The frequencies of the patient group and the control group were 65.3% and 63%, respectively. We found a significant difference between the patient and control groups in terms of the haplotype ratios of c.388A>G and c.521T>C (p = 0.036).

Conclusions

We found a significant relationship between the c.334T>G variant in OATP1B3 and DAA-related side effects in hepatitis C patients.

## Introduction

Hepatitis C virus (HCV) infection is a serious health problem affecting approximately 71 million people worldwide [1]. Three structural proteins (core, E1, and E2) and seven nonstructural proteins (p7, NS2, NS3, NS4A, NS4B, NS5A, and NS5B) are encoded by the HCV genome [2]. Direct-acting antivirals (DAAs) are agents that act on nonstructural proteins of the HCV genome and disrupt viral replication [2]. LDV, OBV, SOF, DSV, PTV, and RTV are DAAs. DAAs include the following: SOF, an NS5B nucleotide polymerase inhibitor; DSV, an NS5B nonnucleoside polymerase inhibitor; PTV, an NS3/4A protease inhibitor; RTV, a CYP3A4 inhibitor; and LDV and OBV, NS5A inhibitors [3].

Compared to interferon-based regimens (pegylated interferon (2a and 2b)), DAA therapy is well tolerated and highly effective in the majority of patients. The most common side effects, which are seen in less than 10% of patients, are diarrhea, headache, fatigue, and nausea. A very small number of patients receiving DAA therapy also require second-line antiviral therapy. The use of ribavirin (RBV) is still recommended, especially for patients with advanced liver disease [1].

When HCV patients are treated with telaprevir or boceprevir, which are first-generation protease inhibitors, the risk of drug-drug interactions (DDIs) is up to 49% [4]. There are fewer DDI-related next-generation NS5A inhibitors. However, side effects may be related to pathways such as P-glycoprotein, CYP3A, organic anion-transporting polypeptide (OATP)1B1, OATP1B3, and OATP2B1, as well as CYP2C19, CYP2C9, CYP2D6, and UGT1A1 [5]. The risk of DDI is greater when patients with advanced cirrhosis are treated with a combination of two or more DAAs [6].

A member of the membrane solute transporter family, OATPs (OATPs in humans, Oatps in rodents, and the gene symbol solute carrier organic anion transporter family (SLCO)), mediates the sodium-independent transport of a range of endogenous and exogenous substances. The HUGO Gene Nomenclature Committee decided to retain the stem gene symbol “solute carrier superfamily” (SLC), create the new symbol “SLCO” for gene classification, and retain the symbol “OATP” for the corresponding protein nomenclature [7].

Under normal physiological conditions, OATP1B1 is expressed almost exclusively in the sinusoidal membrane of hepatocytes, whereas OATP1B3 is also expressed at a much lower level in other tissues, such as the colon, testis, and prostate [7]. OATP1B1 and OATP1B3 in the liver play important roles in the hepatic uptake of various anionic drugs, such as DAAs.

Numerous OATP variations have been found, and they have been linked to notable alterations in the pharmacokinetic profiles of drug substrates. Genetic variations in OATP1B1 or OATP1B3 affect the systemic concentrations of these drugs [8]. While *5/*5, *5/*15, and *15/*15 are high-risk genotypes showing adverse reactions to statins, *1a/*5, *1a/*15, and *1b/*15 genotypes are medium-risk, and *1a/*1a, *1a/*1b, and *1b/*1b genotypes are low-risk genotypes.

For example, variation (c.521T>C) in the SLCO1B1 gene encoding OATP1B1 decreases the ability of OATP1B1 to transport active simvastatin acid from portal circulation into the liver, resulting in markedly increased plasma concentrations of simvastatin acid and an enhanced risk of simvastatin-induced myopathy. SLCO1B1 polymorphism also affects the pharmacokinetics of many other, but not all (fluvastatin), statins and that of the antidiabetic drug repaglinide, the antihistamine fexofenadine, and the endothelin A receptor antagonist atrasentan [9].

In fact, 388A>G and 521T>C form four different haplotypes: *1A (388A/521T), *1B (388G/521T), *5 (388A/521C), and *15 (388G/521C). The OATP1B1*5 and *15 genotypes cause markedly reduced hepatic uptake, which increases systemic exposure to substrates [10], whereas OATP1B1*1B has been associated with reduced systemic exposure to substrates [11]. Reduced transport of OATP1B1 and OATP1B3 may increase the risk of adverse drug reactions. Therefore, it is necessary and very important to investigate the interactions between DAAs and OATP1B1/OATP1B3 [8].

This study aims to assess whether genetic variations in the liver transporters OATP1B1 and OATP1B3 influence the incidence and severity of DAA-related side effects in hepatitis C patients.

This article was initially published as a preprint on Research Square on June 30, 2024 (https://doi.org/10.21203/rs.3.rs-4578225/v1).

## Materials and methods

Study population and design

In this study, 199 chronic HCV patients receiving DAA treatment and 162 healthy individuals were included by random sampling method. Blood samples for the study were collected at the Department of Gastroenterology, Mersin University Hospital, Turkey. All the patients were informed about the study protocol and provided informed consent. The study protocol was approved (2016/194) by the Mersin University Ethics Committee. All subjects underwent a comprehensive medical examination, routine blood examination, and biochemical examination. We collected basic demographic information from patients based on information recorded in the medical records system.

The control group consisted of people in a similar age group without chronic liver disease and nonpregnant, nonbreastfeeding women and males. Throughout the course of treatment and follow-up, patients were seen at the outpatient clinic on a regular basis. At every appointment, patients received a medical examination as well as biochemical, serological, and virological testing.

Inclusion and exclusion criteria

Patients with positive anti-HCV and HCV-RNA test results, diagnosed with chronic HCV infection, treated with DAAs according to national guidelines for the management of HCV infection, and aged between 18 and 90 years were included in the study. Patients with coinfections, those under 17 or over 90 years old, and women who were pregnant or breastfeeding were excluded from the study.

Treatment regimens

We collected blood samples from patients diagnosed with HCV who were treated with DAA between July 2016 and December 2019. A total of 63 patients were treated with a combination of RTV-boosted PTV, OBV, and DSV, while 136 patients were treated with LDV and SOF with or without RBV. Moreover, 63 patients were treated with a combination of PTV, OBV, and DSV boosted by RTV for 12 weeks. DSV (250 mg/day, twice daily), RTV (100 mg/day), PTV (150 mg/day), and OBV (25 mg/day) were administered. Additionally, 200 mg/day of RBV was administered to only three patients. RBV was discontinued if the hemoglobin value decreased by 2 g/dL or more in less than four weeks or if the hemoglobin value decreased below 10 g/dL.

Patients were invited to monthly follow-up visits as long as they had no additional complaints. Serum HCV RNA, blood biochemistry, hemograms, and side effects were recorded at the patients’ monthly checkups. These evaluations were conducted at four, 12 (end of treatment), and 24 weeks (to assess the sustained viral response 12 weeks after treatment).

A total of 126 patients received 90 mg of LDV and 400 mg of SOF in a fixed-dose combination tablet once daily with or without RBV, according to the physicians’ suggestions. RBV was initially administered orally at a low dose of 600 mg/d (twice daily), which was then increased to a maximum dose of 1,000-1,200 mg/d if the patient tolerated it. The proposed treatment duration was 24 weeks.

Side effect classification

The patient group was surveyed regarding drug side effects and classified into five groups based on their reported symptoms. Group 0 included patients with no side effects, while Group 1 consisted of those experiencing itching. Group 2 was for patients reporting fatigue, and Group 3 included those with skin and mucosal lesions, such as dryness, redness, blistering of the skin, hair loss, sweating, dry mouth, cracked tongue, and sores inside the mouth. Finally, Group 4 covered a range of other side effects, including nausea, loss of appetite, trembling hands, hot flashes, depression, nervousness, arthralgia, headache, dyspnea, vision loss, and cough.

Blood samples

All blood samples for genotyping were taken before initiating treatment. Whole blood was collected into vacuum tubes containing EDTA as an anticoagulant for genomic DNA analysis and stored at -20°C until DNA isolation. Genomic DNA extraction was performed according to the manufacturer’s protocols using the Purelink Genomic DNA Isolation Kit (Invitrogen, Waltham, Massachusetts, USA) based on the spin column method.

Analysis of SLCO1B1 and SLCO1B3 variants was performed with polymerase chain reaction-restriction fragment length polymorphism (PCR-RFLP). The primer pairs used for the amplification of the SLCO1B1 gene and the SLCO1B3 gene and the results of the PCR-RFLP evaluation method are summarized in Table 1.

**Table 1:** Primer pairs and PCR-RFLP evaluation method used for the amplification of OATP1B1 and OATP1B3 OATP, organic anion-transporting polypeptide; PCR-RFLP, polymerase chain reaction-restriction fragment length polymorphism

Variation		Primer		Temperature of annealing	Restriction endonuclease	PCR products
OATP1B1	c.388 A>G variation (rs2306283)	Primer F	5’-CTGTGTTGTTAATGGGCGAA-3’	57.0°C	TaqI	A allele: 159, 247 bp; G allele: 23, 136, 247 bp
		Primer R	5’-GGGGAAGATAATGGTGCAAA-3’			
	c.521T>C variation (rs4149056)	Primer F	5’-TTGTCAAAGTTTGCAAAGTG-3’	52.0°C	Hin6I	T allele: 209 bp; C allele: 21,188 bp
		Primer R	5’-GAAGCATATCCATGAGC -3’			
OATP1B3	c.334T>G variation (rs4149117)	Primer F	5’-GAAGGTACAATGTCTTGGGC-3’	51.0°C	AluI	T allele: 86, 253 bp; G allele: 126, 213 bp
		Primer R	5’- CTCTCAAAAGGTAACTGCCC -3’			
	699 G>A variation (rs7311358)	Primer F	5’-ATGATTACATTCCCTGGATC-3’	55.0°C	RsaI	G allele: 61, 242 bp; A allele: 28, 275 bp
		Primer R	5’-ACTATCATGGTACCTTGTTC-3’			

Statistical analysis

Statistical analysis was performed using Statistica, Version 13.5.0.17 (TIBCO Software Inc., Palo Alto, California, USA). Categorical variables were analyzed with the chi-square test and are expressed as frequencies (%).

The differences in allelic and genotypic frequencies between the groups, as well as the Hardy-Weinberg equilibrium for the c.388A>G variant (rs2306283), c.521T>C variant (rs4149056), c.334T>G variant (rs4149117), and 699A>G variant (rs7311358) genotype distributions, were evaluated.

Independent sample t-tests were used to compare continuous variables between two independent groups, and one-way ANOVA was used for comparisons among more than two groups. The results were considered statistically significant if p < 0.05.

## Results

The study included 199 chronic HCV patients and 162 healthy individuals. The average age of the patients was 61; 108 (54.2%) of them were women, and 91 (45.8%) were men. The average age of the controls was 58 years, and there were 59 (36.4%) men and 103 (63.6%) women. There was no significant difference between the groups in terms of age or sex. A total of 183 of 199 patients had genotype 1 HCV, and the mean baseline HCV RNA concentration was 2,640,442 IU/mL. A total of 136 (68.3%) of these patients used LDV/SOF, 63 (31.7%) patients used ombitasvir/paritaprevir/ritonavir ± dasabuvir (OPrD), and 56 (28.1%) patients had cirrhosis. In 44 patients, RBV was used in combination with these drugs due to indications. Thirty-six (81.8%) of these patients used ledipasvir/sofosbuvir (LS), and eight (18.2%) of these patients used RBV in combination with OpRD. The HCV-RNA status of all patients became negative at the end of treatment and remained negative at 12 weeks after discontinuation of treatment. The characteristics of the patients are summarized in Table 2.

**Table 2:** Patient characteristics HCV, hepatitis C virus; LDV, ledipasvir; OPrD, ombitasvir/paritaprevir/ritonavir ± dasabuvir; SOF, sofosbuvir

Patient characteristics	n (%)
Age (years), mean (SD)	61 (15.05)
Gender (female/male)	108/91 (54.2/45.8)
Genotype 1/2/3/4 (n)	183/3/5/2*
HCV RNA (mean, IU/mL)	2,640,442
LDV/SOF (n) (%)	136 (68.3)
OPrD (%)	63 (31.7)
Ribavirin + (LDV/SOF/OPrD) (%)	36/8 (81.8/18.2)
Liver cirrhosis	56 (28.1)

All 199 patients tolerated the medication, and none of them discontinued the drug. While 146/199 (73.4%) of the patients experienced no side effects, 53 (26.6%) experienced side effects. Skin lesions (redness, skin rash, skin dryness, etc.) were observed in 19 (9.5%) of the 53 patients with side effects, fatigue in 18 (9%), itching in 11 (5.5%), and other side effects in five (2.5%) patients. A decrease in hemoglobin was observed in three patients who received RBV, but the hemoglobin concentration in none of them decreased to less than 10 g/dl. There was no difference between the drugs used in terms of side effects: 39/136 (28.9%) in the LS group versus 14/63 (28%) in the OPrD group (Table 3).

**Table 3:** Relationship between drugs and side effects p = chi-square test LDV, ledipasvir; OPrD, ombitasvir/paritaprevir/ritonavir ± dasabuvir; SOF, sofosbuvir

Medications	Side effects (n/%)	p
LDV/SOF	39 (28.9)	p = 0.386
OprD	14 (23.0)	

While no side effects were observed in 146 patients, side effects were observed in 53 patients: skin and mucosal lesions were observed in 19 patients (36%), fatigue in 18 patients (34%), itching in 11 patients (20.5%), and other side effects (nausea, loss of appetite, tremors in hands, hot flashes, depression, irritability, joint pain, headache, shortness of breath, vision loss, and cough) in five patients (9.5%).

Distribution of OATP variants

In the OATP1B1 c.388A>G variant, the frequencies of AA, AG, and GG genotypes are 40.2%, 37.7%, and 22.1%, respectively. The genotype frequencies of the c.521T>C variant were as follows: TT, 76.8%; TC, 18.7%; and CC, 4.5%. OATP1B3 was detected in the following patient groups: the c.699G>A variant GG genotype, 4.5%; the AG genotype, 19.1%; the AA genotype, 76.4%; the c.334T>G variant TT genotype, 6.5%; the TG genotype, 28.1%; and the GG genotype, 65.3%. Among the variants evaluated, there was a significant association between having the c.334 T>G and side effects (p = 0.030). The frequency of the 334TT variant was 199/130 (65.3%) in the patient group and 162/102 (63%) in the control group, indicating a balanced distribution (Hardy-Weinberg equilibrium p = 0.05). When the OATP variants we examined were evaluated according to treatment, there was no significant difference in side effects between the two groups. There was no relationship between OATP variants and side effects in the LS group. The risk of side effects of the OATP1B3 c.334T>G variant for OPrD increased by 5.89 (1.62-21.29) in carriers of the 334TT genotype compared to carriers of the 334TG genotype (p = 0.018).

Relationships between side effects and variants

The OATP1B1c.388 A>G variations: Among the 199 patients, 80 had the 388AA genotype, 75 had the 388AG genotype, and 44 had the 388GG genotype; the incidence of side effects was 20/80 (25%), 26/75 (34.6%), and 7/44 (15.9%), respectively. No significant relationship was found between the occurrence of side effects and OATP1B1 c.388 A>G (p = 0.503). There was no relationship between genotype and the type of side effect in those with side effects (p = 0.075). The relationship between side effects and genotypes is given in Table 4.

**Table 4:** Genotype and side effect relationship p = chi-square test An asterisk (*) indicates a significantly higher rate (p < 0.05). OATP, organic anion-transporting polypeptide

OATPs distribution		Genotype	Side effect					Total	p
			0	1	2	3	4		
OATP1B1	c.388A>G	AA	60	2	9	7	2	80	0.075
		AG	49	6	8	9	3	75	
		GG	37	3	1	3	0	44	
	c.521T>C	TT	112	6	16	14	5	153	0.379
		TC	29	4	1	3	0	37	
		CC	5	1	1	2	0	9	
OATP1B3	c.334T>G	TT	9	0	4	0	0	13	0.030*
		TG	34	5	7	9	1	56	
		GG	103	6	7	10	4	130	
	c.699G>A	GG	6	0	3	0	0	9	0.825
		GA	29	3	3	1	2	38	
		AA	111	8	12	18	3	152	

OATP1B1 c.521T>C Variations

Among the 199 patients, 153 had the 521TT genotype, 37 had the 521TC genotype, and nine had the 521TT genotype; the incidence of side effects was 41/153 (26.7%), 8/37 (21.6%), and 4/9 (44.4%), respectively. However, no significant relationship was found between the occurrence of side effects and the OATP1B1 c.521A>G (p = 0.379). There was no relationship between genotype and the type of side effect in those with side effects (p = 0.295).

OATP1B3 334 T>G Variations

Among the 199 patients, 13 had the TT genotype, 56 had the TG genotype, and 130 had the TT genotype; the incidence of side effects was 4/13 (30.7%), 22/56 (39.2%), and 27/130 (20.7%), respectively. A significant relationship was found between the occurrence of side effects and OATP1B3 c.334T>G (p = 0.030). We found that carrying the T allele increased the risk of side effects while carrying the G allele decreased the rate of side effects. On the other hand, there was no relationship between genotype and the type of side effect in those with side effects (p = 0.127).

OATP1B3 699 A>G Variations

Among the 199 patients, nine had the 699AA genotype, 38 had the 699AG genotype, and 152 had the 699GG genotype; the incidence of side effects was 3/9 (33.3%), 9/38 (23.6%), and 41/152 (26.9%), respectively. No significant relationship was found between the occurrence of side effects and the OATP1B3 gene 699 A>G (p = 0.825). Among those with side effects, no relationship was found with genotype according to the type of side effect (p = 0.093).

The allele frequencies of the 388A>G, c.521T>C, c.334T>G, and c.699G>A variants of the OATP gene did not differ significantly between the control and patient groups (p > 0.05). The relationship between side effects and genotypes is given in Table 4.

Haplotype analysis

The allele/genotype combinations of the OATP1B1 haplotypes are shown in Table 5. We found a significant difference between the patient and control groups in terms of the haplotype ratios of c.388A>G and c.521T>C (p = 0.036). Accordingly, while the 1b/1b ratio was greater in the patient group, we observed a greater 1a/1b ratio in the control group ( p <0.05) (Table 6).

**Table 5:** OATP1B1 allele/genotype combinations

	c.521T>C			
c.388A>G		TT	TC	CC
	AA	*1a/*1a	*1a/*5	*5/*5
	AG	*1a/*1b	*1a/*15 or *1b/*5	*5/*15
	GG	*1b*/1b	*1b/*15	*15/*15

**Table 6:** OATP1B1 c.388A>G (rs2306283) and c.521T>C (rs4149056) allele/genotype combinations and haplotype frequencies of 388A>G and 521T>C in patient and control groups p = chi-square test An asterisk (*) indicates a significantly higher rate (p < 0.05).

Haplotype	Patient		Control		p
	n	%	n	%	
*1a/*1a	63	31.7	44	27.2	0.352
*1a/*5	12	6	10	6.2	0.955
*5/*5	5	2.5	4	2.5	0.979
*1a/*1b	55	27.6	70	43.2	0.002*
*1a/*15 or *1b/*5	18	9	12	7.4	0.575
*5/*15	2	1	5	3.1	0.154
*1b/*1b	35	17.6	14	8.6	0.014*
*1b/*15	7	3.5	2	1.2	0.166
*15/*15	2	1	1	0.6	0.686

While the *1b/*1b ratio was higher in the patient group compared to the control group, the *1a/*1b ratio was lower (p < 0.05). We did not find a significant difference in haplotype frequencies according to sex in the patient group (p = 0.376). There was no difference between the mean age and haplotype (p = 0.170). No significant relationship was found between the haplotypes and side effects (p = 0.136). The relationships between the study patients’ age, sex, side effect characteristics, and haplotype groups are summarized in Table 7.

**Table 7:** Demographic characteristics of patients according to haplotype group ^a^ Chi-square test ^b^ One-way ANOVA

Characteristics		*1a/*1a	*1b/*1b	*1a/*1b	*1b/*15	*1a/*15 and *1b/*5	*15/*15	*1a/*5	*5/*5	*5/*15	p
Subjects (N, %)		63 (31.7)	35 (17.6)	55 (27.6)	7 (3.5)	18 (9.0)	2 (1.0)	12 (6.0)	5 (2.5)	2 (1.0)	
Gender	Female (N, %)	34 (31)	14 (13.0)	36 (33.3)	4 (4.6)	9 (8.3)	1 (0.9)	7 (6.5)	1 (0.9)	1 (0.9)	0.376^a^
	Male (N, %)	29 (31)	21 (23.1)	19 (20.9)	2 (2.2)	9 (9.9)	1 (1.1)	5 (5.5)	4 (4.4)	1 (1.1)	
Age (years) (mean (SD))		53.44 (18.02)	58.63 (14.48)	54.98 (16.97)	67.44 (8.66)	52.67 (17.14)	41.33 (13.86)	56.59 (13.65)	51.33 (20.38)	51.71 (16.59)	0.170^b^
Side effect	Yes (N, %)	16 (30.2)	5 (9.4)	20 (37.7)	1 (1.9)	4 (7.5)	1 (1.9)	3 (5.7)	1 (1.9)	2 (3.8)	0.136^a^
	No (N, %)	47 (32.2)	30 (20.5)	35 (21.0)	6 (4.1)	14 (9.6)	1 (0.7)	9 (6.2)	4 (2.7)	0	

## Discussion

Hepatic drug transporters play an important role in the excretion process of drugs in the liver, which is the main organ where the first-pass effect occurs. OATPs are encoded by SLCO genes and are members of the SLC that mediate the uptake of substrate influx. OATPs mediate the transport of various exogenous and endogenous substances independently of sodium and ATP [7,12]. Genetic variations can result in drug plasma concentrations varying by up to 600 times in individuals with the same body weight receiving the same standard dose and are a major contributor to the significant interindividual variability in therapeutic response and toxicity observed in the majority of patient populations [13].

Of the 199 individuals with hepatitis C in our study, 53 (26.6%) experienced adverse effects. Of the 53 patients with side effects, 19 (35.9%) had skin lesions, 18 (34%) had fatigue, 11 (20.7%) had itching, and five (9.4%) had nausea. The c.334T>G variation and side effects were significantly correlated (p = 0.030 for all).

The minor allele frequency (MAF) of the c.388A>G variant is 42.8% [14], and the c.388A>G variant does not significantly impact the pharmacokinetics, response, or toxicity of drugs. In contrast, other OATP1B1 variants, except for c.388 A>G, have important clinical significance. In our study, we found that the c.388A>G variant had a 41% MAF in the patient group and a 37.3% MAF in the control group. The c.521T>C variant has a 14.7% MAF and, when expressed in cells in vitro, causes decreased expression at the plasma membrane and decreased transport activity due to a greater degree of protein phosphorylation [15]. The expression of 388A>G was significantly greater than that of wild-type 388A>A, while there was no difference in protein expression between 521T>C and wild-type 521T>T [16]. Since we did not evaluate c.388A>G or c.521T>C expression in our study, we cannot evaluate this issue.

Variations that reduce the activity of OATP1B1, which plays an important role in the transport of statins from the portal vein to liver cells, may lead to decreased statin levels in the liver and increased statin levels in plasma. As a result, c.521T>C may increase the risk of myopathy and rhabdomyolysis [17].

Plasma levels of the anti-HIV drug atazanavir are greater in patients carrying the c.521T>C variant and tapering of the drug has been recommended to avoid side effects [18]. The SEARCH Collaborative Group [19] reported the relationship between the risk of simvastatin-induced myopathy and the OATP1B1 c.521T>C variation. The 521C allele has a prevalence of 8-22% in Caucasian populations [20], 8-19% in East Asian populations [18], and 15% in the Netherlands [21]; however, its prevalence in African and African-American populations is only 1-8% [20]. In our study, we found that the frequency of the c.521T>C variant allele was 13.6% in the control group, while it was 13.8% in the patient group (p = 0.926). Our finding is consistent with the report of Romaine et al. [20].

We also investigated whether OATP1B1 (c.388 A>G, c.521T>C) and OATP1B3 (c.334 T>G, c.699 G>A) were associated with the side effects of OATP as a carrier in hepatitis C patients. Since plasma concentrations of DAAs were not evaluated in our study, it is unknown whether the 388AG variant causes increased plasma concentrations of DAAs.

OATP1B3 is a drug transporter that is highly expressed in the human liver [22]. Many genetic variants of SLCO1B3 have been associated with reduced transport activity or OATP1B3 expression in vitro. Among these, the most clinically relevant variations are c.334T>G and c.699G>A, and the frequencies of these variations are 84.2% and 85.3%, respectively [23]. We found a significant correlation between the OATP1B3 c.334T>G variation and side effects in the patient group (p = 0.030). Accordingly, carrying the T allele increases the rate of side effects, while carrying the G allele decreases the rate of side effects.

Pasanen et al. reported that the low-activity haplotypes *5 (388A/521C) and *15 (388G/521C) have a combined frequency of approximately 15-20% in Europeans, 10-15% in Asians, and 2% in sub-Saharan Africans. The frequency of the *1B (388G) haplotype is approximately 26% in Europeans, 39% in South/Central Asians, 63% in East Asians, and 77% in sub-Saharan Africans [24]. Kameyama et al. reported that the c.388A>G+c.521T>C haplotype and c.521T>C variation were associated with decreased transport activity [11]. It is unknown whether the increase in OATP1B1 transport activity caused by the c.388A>G variant leads to increased plasma concentrations of DAAs. In our study, when we compared those who developed side effects with those who did not develop side effects, we could not find a relationship between the c.388A>G variant and genotype (p = 0.075).

The presence of the c.388A>G variant varies significantly between ethnic groups and is detected in 77.8% of African individuals [25]. The OATP1B1*5 and OATP1B1*15 haplotypes showed greatly reduced transport activities for all tested substrates, such as E17βG, E3S, atorvastatin, docetaxel, methotrexate, and pravastatin.

OATP1B1 and OATP1B3, which mediate the transport of many drugs, have overlapping substrate properties. These substances include bile salts, bilirubin, coproporphyrins, hormones such as thyroxine, triiodothyronine, and their conjugates, leukotriene C4, and cyclic and linear peptides [26]. OATP1B3 carries special substrates, such as cholecystokinin octapeptide, testosterone, digoxin, epicatechin gallate, epigallocatechin gallate, paclitaxel, ouabain, dioscin, and indocyanine green. However, these proteins are not carried by OATP1B1 [27-36]. It has been shown that c.334T>G has no significant effect on the pharmacokinetics of most of the E17bG, taurocholate, cholecystokinin octapeptide, glibenclamide, glipizide, and paclitaxel substrate drugs [37-39]. Yamakawa et al. showed that the clearance of imatinib was significantly greater in patients with the OATP1B3 334GG genotype than in those with the OATP1B3 334TT genotype [40]. Park et al. reported that c.699G>A has a moderate effect on paclitaxel transport [38], and Yang et al. reported that c.699G>A has a significant effect on glibenclamide and glipizide transport [39]. Schwarz et al. reported that c.699G>A caused a moderate decrease in CCK-8 transport [41]. In vitro functional studies have shown that c.699G>A has a moderate effect, whereas c.334T>G has no significant effect on the function of OATP1B3. Allele frequencies of both the 334T>G and 699G>A variations were reported to be 73%, and both variations have thus far been reported to have no major influence on the function of OATP1B3 [26].

In our study, we found that the allele frequencies of the 334T>G and 699G>A variants were 79.4% and 85.9%, respectively. Tsujimoto et al. reported in their study of hemodialysis patients that c.334T>G and c.699G>A could increase the digoxin plasma concentration-to-dose ratio and could be useful for adjusting its dosage, but this increase was not statistically significant [42]. Smith et al. reported that the pharmacokinetics of paclitaxel in Caucasian cancer patients were not significantly associated with the SLCO1B3 genotype [43].

In general, the expression of OATP1B1 and OATP1B3, which are specific to the liver, tends to decrease in hepatocellular carcinomas [44]. Rotor syndrome is an autosomal recessive disease, a rare and benign hyperbilirubinemia. Rotor syndrome results in abnormally short, nonfunctional OATP1B1 and OATP1B3 proteins or the absence of these proteins [45]. In our study, we found a significant relationship between the T allele of OATP c.334T>G and side effects (p = 0.030). The rate of side effects was greater in those carrying the OATP 334T allele.

In the study by Li et al., the most frequently reported side effects during multiple-dose administration of LDV/SOF were proteinuria and oropharyngeal pain [46]. During SOF/velpatasvir administration, an increase in alanine aminotransferase levels was the most frequently reported side effect. Drugs such as gemfibrozil, cyclosporin A, rifampicin, and RTV, which are OATP1B1 and OATP1B3 inhibitors, may cause major side effects such as myopathy when they are administered with lipid-lowering statins, which are OATP substrates [27,47-49]. In our study, the most common side effects were fatigue and skin symptoms, which were observed in 34% and 36%, respectively.

In a study of patients with compensated liver cirrhosis and genotype 1, 24 weeks of treatment with the OPrD regimen and RBV combination was not superior to 12 weeks of treatment. Additionally, the 24-week treatment duration was associated with more side effects, including fatigue, headache, and nausea, compared to the 12-week regimen [50]. In our study, the most common side effects were skin and mucosal lesions, fatigue, and itching, which were observed in 36%, 34%, and 20.5%, respectively.

Örmeci et al. reported that in HCV-infected patients treated with DAAs, the most common side effects were anemia, itching, fatigue, and headache [51]. Although the most common side effect in their study was anemia (82.44%), skin lesions, fatigue, and itching were the most common side effects.

The current study is the first to investigate and demonstrate the association between adverse events and OATP variants (especially OATP 334T>G) in hepatitis C patients treated with DAAs. However, more comprehensive studies are needed to evaluate which variant is more effective, which variant is associated with side effects, and whether it affects drug efficacy. OATP1B1 c.388A>G and c.521T>C are the most commonly investigated variations. This study investigated the frequencies of four common polymorphic variations and the relationships between the side effects of these drugs among those diagnosed with HCV and those receiving drug therapy in Turkey.

This study has several limitations. First, the sample size was relatively small. Additionally, the relationship between drug levels, side effects, and treatment outcomes was not assessed. Nevertheless, our study is the first to report a potential relationship between DAAs and OATP variants.

## Conclusions

DAAs are the standard of care for the treatment of HCV infection, and the failure rate of DAAs in clinical practice is low. DAA regimens such as RBV/SOF, LDV/SOF, and simeprevir/SOF have higher failure rates than other DAAs. The implications of actual data on approved HCV treatment regimens are extremely important in clinical practice. Clinical studies allow us to evaluate the effectiveness and safety of regimens in real-life patients. We examined OATP1B1 c.388A>G and c.521T>C variations and OATP1B3 c.334T>G and c.699G>A variations in hepatitis C patients treated with DAAs and controls. We found a significant association between the c.334T>G variant in OATP1B3 and DAA-related side effects in hepatitis C patients.

The future of medicine is going through many changes that support advanced diagnostics of diseases, early detection of genetic predisposition to diseases, gene therapy, and pharmacogenomics for personalized medicines. Since the general aim in the treatment of diseases is to optimize the medical care and results of each individual, ensuring genotype-phenotype correlation allows patients to apply a treatment program that is more effective and shorter and minimizes side effects.
