# Correction: The Impact of Organic Anion-Transporting Polypeptide (OATP) Variants on the Side Effects of Direct-Acting Antivirals in Hepatitis C Patients

**DOI:** 10.7759/cureus.c442

**Published:** 2026-05-29

**Authors:** Zuhal Altintas, Engin Altintas

**Affiliations:** 1 Medical Genetics, Faculty of Medicine, Mersin University, Mersin, TUR; 2 Gastroenterology and Hepatology, Faculty of Medicine, Mersin University, Mersin, TUR

This article has been corrected to transpose the G allele and A allele in the final row of Table 1.

